# 基于固相萃取-高效液相色谱-串联质谱测定孕妇尿液中8种邻苯二甲酸酯代谢物及新生儿出生结局评价

**DOI:** 10.3724/SP.J.1123.2023.12032

**Published:** 2025-01-08

**Authors:** Zihao WANG, Mengfei XU, Beini LI, Ping WU, Wei WU

**Affiliations:** 湖北中医药大学检验学院, 湖北 武汉 430065; Hubei University of Chinese Medicine, Wuhan 430065, China

**Keywords:** 高效液相色谱-串联质谱, 邻苯二甲酸酯代谢物, 孕妇尿液, 出生结局, high performance liquid chromatography-tandem mass spectrometry (HPLC-MS/MS), phthalate metabolites (mPAEs), urine samples of pregnant women, birth outcomes

## Abstract

邻苯二甲酸酯(PAEs)是一种环境内分泌干扰物,广泛存在于日常生活中,并可通过多种途径进入机体。孕妇作为一类特殊人群,若暴露于PAEs,将会对子代的生长发育造成不良影响。研究建立了固相萃取-高效液相色谱-串联质谱(SPE-HPLC-MS/MS)同时检测孕妇尿液中8种PAEs代谢物(mPAEs)的分析方法。尿液样品经*β*-葡萄糖苷酶酶解后,采用Bond Elut Plexa固相萃取柱净化,样品经洗脱、浓缩、复溶后,进行HPLC-MS/MS分析。采用Agilent Eclipse Plus C18色谱柱(100 mm×3 mm, 3.5 μm)进行分离,以0.1%乙酸水溶液和0.1%乙酸乙腈作为流动相进行梯度洗脱,在多反应监测(MRM)模式下检测,内标法定量。 结果表明,8种mPAEs在0.1~200 ng/mL范围内线性关系良好,检出限为0.015~0.048 ng/mL,定量限为0.050~0.160 ng/mL。在低、中、高3个加标水平(1、10、50 ng/mL)下,8种mPAEs的加标回收率为80.2%~99.7%。采用此方法对鄂州市妇幼保健院497名孕妇尿液中的8种mPAEs水平进行检测,结果表明,497名孕妇广泛暴露于PAEs,其中邻苯二甲酸单丁酯(MBP)的含量最高,中位数水平为104.46 ng/mL;邻苯二甲酸单苄酯(MBzP)的含量最低,中位数水平为0.22 ng/mL。同时,研究还对新生儿的出生结局进行了评估,线性回归模型表明,孕妇尿液中的邻苯二甲酸单乙酯(MEP)每增加一个自然对数(ln)水平,孕周减少0.11周(95%置信区间(CI): -0.18~-0.03);孕妇尿液中的邻苯二甲酸(2-乙基-5-氧己基)酯(MEOHP)和邻苯二甲酸单(2-乙基己基)酯(MECPP)每增加一个ln水平,新生儿出生体重分别降低39.28 g (95%CI: -76.48~-2.09)和39.62 g (95%CI: -73.73~-5.52)。该方法操作简便,检出限低,具有较高的准确度和精密度,通过此方法测定了孕妇尿液中的8种mPAEs,并结合新生儿的出生结局发现,孕期PAEs暴露会影响新生儿的生长发育。

## Abstract (en)

Phthalates (PAEs) are endocrine-disrupting chemicals that are widely present in everyday life and enter the human body through various pathways. The release of PAEs into the environment through pathways that include leaching, evaporation, abrasion, and the use of personal care products exposes humans to PAEs via ingestion, inhalation, and dermal absorption. Pregnant women, as a particularly vulnerable population, risk adverse newborn growth and development when exposed to PAEs. While the concentrations of PAEs in urine reflect recent exposure levels in humans, urinary levels of phthalate metabolites (mPAEs) are commonly used as biomarkers of internal exposure owing to the relatively short biological half-lives of PAEs (<24 h). In this study, we developed a solid-phase extraction-high performance liquid chromatography-tandem mass spectrometry (SPE-HPLC-MS/MS) method for simultaneously detecting eight mPAEs in the urine of pregnant women. Urine samples were enzymatically hydrolyzed with *β*-glucosidase and then purified using the Bond Elut Plexa SPE column, with subsequent elution, concentration, and redissolved performed prior to HPLC-MS/MS. Separation was achieved using an Agilent Eclipse Plus C18 column (100 mm×3 mm, 3.5 μm), with gradient elution performed using 0.1% acetic acid aqueous solution and 0.1% acetic acid acetonitrile as mobile phases. Multiple reaction monitoring (MRM) mode was used for detection, with quantification performed using the internal-standard method. Good linearities were obtained in the range of 0.1-200 ng/mL for the eight mPAEs, with limits of detection (LODs) and quantification (LOQs) of 0.015-0.048 and 0.050-0.160 ng/mL, respectively. The eight mPAEs exhibited recoveries of 80.2%-99.7% at three spiked levels (1, 10, and 50 ng/mL). This method was subsequently used to analyze the eight mPAEs levels in urine samples of 497 pregnant women from the Ezhou Maternity and Child Health Care Hospital. The participants exhibited widespread exposure to PAEs, with monobutyl phthalate (MBP) showing the highest median level of 104.46 ng/mL, and monobenzyl phthalate (MBzP) showing the lowest (0.22 ng/mL). In addition, this study assessed neonatal birth outcomes. Linear regression modeling revealed that gestational age decreased by 0.11 weeks (95% confidence interval (CI): -0.18--0.03) for every natural-log (ln) increase in the level of monoethyl phthalate (MEP) in urine samples of pregnant woman. Moreover, the birth weight decreased by 39.28 g (95% CI: -76.48--2.09) and 39.62 g (95% CI: -73.73--5.52), for every ln increase in mono(2-ethyl-5-oxohexyl) phthalate (MEOHP) and mono(2-ethylhexyl) phthalate (MECPP) levels, respectively. The developed method is characterized by its simplicity, low LODs, high accuracy, and precision. This study provides clear evidence that PAE exposure during pregnancy negatively affects newborn growth and development by measuring the levels of eight mPAEs in the urine of pregnant women and linking these findings to neonatal outcomes. Further large-scale cohort studies are needed to validate these findings, along with mechanistic studies using animal models or in-vitro systems that elucidate the biological pathways through which mPAEs contribute to adverse birth outcomes.

邻苯二甲酸酯(phthalates, PAEs)是一类合成衍生化学品,常被作为增塑剂和添加剂用于许多常见的家用产品中,是一种普遍存在的环境激素。随着使用年限的延长,PAEs会从塑料材料中释放,并迁移到周围的环境介质中,造成人类长期、持续的暴露^[1⇓⇓-4]^。曹鎏等^[5]^发现,作为特殊群体,孕妇普遍暴露于PAEs。PAEs能够穿透胎盘并影响新生儿的生长发育,因此评估孕妇体内PAEs的水平,对于深入探讨PAEs对后代生长发育的潜在影响具有重大意义。

PAEs经人体暴露、吸收后会在体内迅速代谢为单酯,随后转化为亲水性更强的氧化代谢产物,这些代谢产物主要通过尿液排出体外^[6]^。尿液中的PAEs水平更能反映人体近期的暴露状况,但PAEs的生物半衰期相对较短(<24 h),因此常采用尿液中PAEs代谢物(mPAEs)的水平作为机体内的暴露指标^[7]^。人体尿液成分复杂,且经生物体代谢残留的mPAEs多处于痕量甚至超痕量水平,因此建立高效的富集方法和高灵敏度的检测方法对实现人体内mPAEs的暴露水平评估至关重要^[8]^。

色谱-质谱联用技术兼具色谱的高效分离能力和质谱的高分辨、高灵敏度等特性,已广泛应用于暴露组学研究^[9]^。目前,国内外已有许多关于人体内PAEs残留的检测报道。Fernandez等^[10]^将中空纤维液相微萃取(HF-LPME)与气相色谱-质谱(GC-MS)结合,建立了同时测定成年学生尿液样品中双酚A和8种mPAEs的方法。该方法的线性关系、精密度和检出限均较为理想,适用于尿液样品的痕量分析,但该方法的前处理流程较为繁琐,需要对待测物进行衍生化处理。Tang等^[11]^基于填充纤维固相萃取-气相色谱-质谱(PFSPE-GC-MS)技术,建立了儿童尿液中PAEs的测定方法,该方法的预处理装置能够在5 min内完成12个样品的前处理,但其所采用的纳米纤维固相萃取柱较为昂贵且方法检出限较高(0.1~0.5 ng/mL)。

基于此,本研究建立了固相萃取-高效液相色谱-串联质谱(SPE-HPLC-MS/MS)同时测定孕妇尿液中8种mPAEs的高效、灵敏分析方法。采用SPE作为样品前处理方法,无需其他衍生化反应,使用内标法定量。依托于湖北省鄂州市妇幼保健院建立的出生队列,将该法应用于孕妇尿液中8种mPAEs的检测,同时追踪并评估新生儿的生长发育状况,分析孕期PAEs暴露对新生儿出生结局的影响。

## 1 实验部分

### 1.1 仪器、试剂与材料

Agilent 6460高效液相色谱-三重四极杆质谱联用仪、2 mL螺纹口进样瓶、Bond Elut Plexa固相萃取柱(200 mg/6 mL)(美国Agilent公司);0.45 μm 聚四氟乙烯过滤膜(美国F&H公司); 12管防交叉污染SPE装置(美国Supelco公司); BSA124S电子天平(北京Sartorius有限公司); PHS-3E pH计(上海仪电科学仪器股份有限公司); THZ-22台式恒温振荡器(苏州培英实验设备有限公司);超纯水仪(美国Millipore公司)。

甲醇、乙腈、乙酸乙酯(HPLC级)购自美国Thermo Fisher公司;*β*-葡萄糖苷酶溶液(200 U/mL)、磷酸(纯度≥85.0%)购自美国Sigma-Aldrich公司;氢氧化钠(纯度≥98.0%)、乙酸铵(纯度≥99.0%)购自中国医药集团有限公司。

8种mPAEs标准品:邻苯二甲酸单丁酯(MBP)、邻苯二甲酸单苄酯(MBzP)、邻苯二甲酸(2-乙基-5-羟基己基)酯(MEHHP)、邻苯二甲酸单(2-乙基己基)酯(MEHP)、邻苯二甲酸(2-乙基-5-氧己基)酯(MEOHP)、邻苯二甲酸单乙酯(MEP)、邻苯二甲酸单甲酯(MMP)、邻苯二甲酸单(2-乙基-5-羧基戊基)酯(MECPP),纯度均≥98%,购自美国Accustand公司;8种同位素内标:^13^C_4_-MBP、^13^C_4_-MBzP、^13^C_4_-MEHHP、^13^C_4_-MEHP、^13^C_4_-MEOHP、^13^C_4_-MEP、^13^C_4_-MMP、^13^C_4_-MECPP,内标溶液的质量浓度均为100 μg/mL,购自美国剑桥同位素实验室。

### 1.2 标准溶液的配制

8种mPAEs混合标准溶液的配制:分别准确称取MBP、MBzP、MEHHP、MEHP、MEOHP、MEP、MMP、MECPP各0.1 g,用甲醇溶解,配制成质量浓度为1 mg/mL的单标储备液。移取20 μL各单标储备液于容量瓶中,用甲醇定容,配制成质量浓度为2 μg/mL的混合标准储备液,再用甲醇稀释成系列质量浓度(0.1、0.5、2、10、20、100 ng/mL)的混合标准溶液,置于4 ℃冰箱中保存备用。

8种mPAEs内标混合溶液的配制:分别准确量取100 μL的8种内标溶液,用甲醇稀释成质量浓度为10 μg/mL的内标中间液。使用前准确量取400 μL内标中间液于尖底玻璃试管中,用甲醇配制成质量浓度为400 ng/mL的内标混合溶液,用封口膜封口,于4 ℃冰箱中保存备用。

### 1.3 样本采集

本研究基于湖北省鄂州市妇幼保健院建立的出生队列开展,以2019年之前来院做产检的孕妇作为研究对象。入选者符合以下标准:(1)在鄂州市妇幼保健院建卡且在该院产检和分娩;(2)保留了产检时的尿液样本;(3)孕妇无急、慢性疾病史和家族神经病史;(4)产检期间未服用任何药物。受试者信息采用问卷调查的方式获取,征得孕妇同意后,由本人完成问卷填写。孕妇分娩时,由受过专业训练的护士测量新生儿的身长与体重,并依照孕妇建卡信息计算孕周。采用Epidata 3.1软件对数据进行双录入,并建立数据库。

收集孕晚期(>28周)孕妇的晨尿,取中段尿,将尿液装于聚乙烯材质的离心管中,另取纯水作为现场采样空白,放入冰盒中,避光带回实验室,统一存放于本院实验中心的超低温(-80 ℃)冰箱。在检测前一天将尿液样品从冰箱取出,于4 ℃下解冻。本研究得到湖北中医药大学伦理委员会批准(No. 2018-IEC-010),招募志愿者均签署了知情同意书。

### 1.4 样品前处理

取1.0 mL待测尿液,加入120 μL磷酸溶液(1 mol/L)进行酸化,再分别加入280 μL氢氧化钠溶液(1 mol/L)、250 μL乙酸铵缓冲液(1 mol/L)、40 μL内标混合溶液(400 ng/mL)和60 μL *β*-葡萄糖苷酶溶液(200 U/mL)进行酶解。依次使用1 mL乙腈和1 mL乙酸铵缓冲液冲洗SPE柱,停留3 min,使之以1 mL/min的流速流出;向上述SPE柱中加入1 mL酶解后的样品溶液,混匀后过柱,控制流速为1 mL/min;依次用1 mL乙腈和2 mL乙酸乙酯进行洗脱,收集洗脱液;用真空装置将SPE柱中的残留液体全部抽出,收集抽滤液;将两次收集的所有液体合并,并于50 ℃下氮吹至干,再加入200 μL乙腈复溶,转移至1.5 mL离心管中,涡旋振荡2 min,在10000 r/min下离心5 min,最后取100 μL上清液至自动进样瓶的内插管中,待进样测定。

### 1.5 分析条件

#### 1.5.1 色谱条件

色谱柱:Agilent Eclipse Plus C18(100 mm×3 mm, 3.5 μm);流动相:A相为0.1%乙酸水溶液,B相为0.1%乙酸乙腈溶液。梯度洗脱程序:0~2 min, 90%A~75%A; 2~17 min, 75%A~70%A; 17~19 min, 70%A~50%A; 19~21 min, 50%A~40%A; 21~23 min, 40%A~35%A; 23~25 min, 35%A~30%A; 25~27 min, 30%A~10%A; 27~32 min, 10%A~90%A。流速:0.2 mL/min;进样体积:10 μL;柱温:40 ℃。

#### 1.5.2 质谱条件

离子源:电喷雾电离(ESI)源,正电离模式;扫描模式:多反应监测(MRM)模式;毛细管电压:4 kV;离子源温度:350 ℃;脱溶剂气温度:300 ℃;脱溶剂气流量:8 L/min;碰撞气:氮气。其他质谱参数见表1。

**表1:** 8种mPAEs及8种同位素内标的保留时间和质谱参数

Compound	Retention time/min	Parent ion^*^ (*m/z*)	Daughter ion (*m/z*)	DP/V	CEs/eV
Monomethyl phthalate (MMP)	7.011	179.0	77.0	43	19, 10
Monoethyl phthalate (MEP)	8.653	193.0	77.0	41	7, 7
Mono-(2-ethyl-5-hydroxyhexyl) phthalate (MEHHP)	15.575	297.0	124.0	27	35, 13
Mono-(2-ethyl-5-oxohexyl) phthalate (MEOHP)	16.855	291.0	121.0	27	35, 13
Mono-*n*-butyl phthalate (MBP)	17.210	221.0	77.0	50	11, 8
Mono-benzyl phthalate (MBzP)	19.101	255.0	77.0	27	13, 10
Mono-(2-ethylhexyl) phthalate (MEHP)	27.150	277.0	134.0	65	17, 13
Mono(2-ethyl-5-carboxypentyl) phthalate (MECPP)	27.552	309.0	167.0	27	35, 27
^13^C_4_-MMP	6.933	183.0	79.0	43	22, 10
^13^C_4_-MEP	8.643	197.0	79.0	41	9, 7
^13^C_4_-MEHHP	15.375	297.0	124.0	27	35, 13
^13^C_4_-MEOHP	16.625	295.0	124.0	27	35, 13
^13^C_4_-MBP	17.110	225.0	79.0	50	11, 8
^13^C_4_-MBzP	18.901	259.0	79.0	27	12, 10
^13^C_4_-MEHP	27.050	281.0	137.0	65	17, 13
^13^C_4_-MECPP	27.452	313.0	179.0	27	33, 27

* Quantitative ion; DP: declustering potential; CEs: collision energies.

### 1.6 研究对象基本信息

共有497对母婴完成了问卷调查,497名孕妇完成了尿液采集步骤,以上均录入统计分析数据库。怀孕次数为1次的孕妇占比最多(50.3%),分娩次数为1次的孕妇占比为79.1%,有415名孕妇的分娩方式为剖腹产;孕妇的学历以大学本科及以上(61.6%)居多;孕妇的平均身体质量指数(BMI)为20.74 kg/m^2^,平均年龄为28.66岁,孕期体重平均增加量为16.74 kg,平均孕周为38.75周。新生儿的平均出生体重为3305.88 g,平均身长为50.11 cm。相关信息见表2。

**表2:** 孕妇及新生儿的人口统计学资料

Characteristic	Number of objects
Mother	
Number of pregnancies	
1	250
2-3	200
≥4	47
Number of deliveries	
1	393
≥2	104
Mode of delivery	
C-section	415
Natural labor	82
Education level	
Elementary and junior high school	84
Vocational and high school	107
University and higher	306
BMI/(kg/m^2^) (Mean±SD)^#^	20.74±2.96
Weight gain/kg (Mean±SD)^#^	16.74±5.27
Age/years (Mean±SD)^#^	28.66±3.71
Gestational week/weeks (Mean±SD)^#^	38.75±1.35
Newborn	
Birth weight/g (Mean±SD)^#^	3305.88±430.84
Birth length/cm (Mean±SD)^#^	50.11±1.59

BMI: body mass index; # *n*=497.

### 1.7 实验室样本质量控制

所有样本统一由专职调查员采集并登记,运输过程中确保无污染。对调查问卷及实验室检测数据进行整理,采用EpiData 3.1软件建立数据库,以平行双盲的方式录入数据,并对数据进行一致性检验和详细的逻辑纠错。为降低空白干扰,实验使用的玻璃器皿、枪头和离心管等实验耗材均为一次性用品;塑料离心管均为聚乙烯材质,且在每次使用前分别用纯水和甲醇润洗两遍;玻璃试管在清洗干净后均于450 ℃下烘烤4 h以上。样品前处理流程严格按照既定的实验步骤和条件进行,移液枪和氮吹仪等设备由专人定时进行检查。

## 2 结果与讨论

### 2.1 质谱条件的优化

按照1.5节分析条件,对待测化合物的母离子、子离子和碰撞能量等参数进行优化。先通过HPLC分离和一级质谱全扫描获得每种待测化合物的保留时间和母离子,然后优化碰撞能量获得子离子,最后采用MRM模式对待测化合物进行定性和定量分析。8种mPAEs及8种同位素内标的总离子流色谱图见图1,相应的质谱参数见表1。

**图1:**
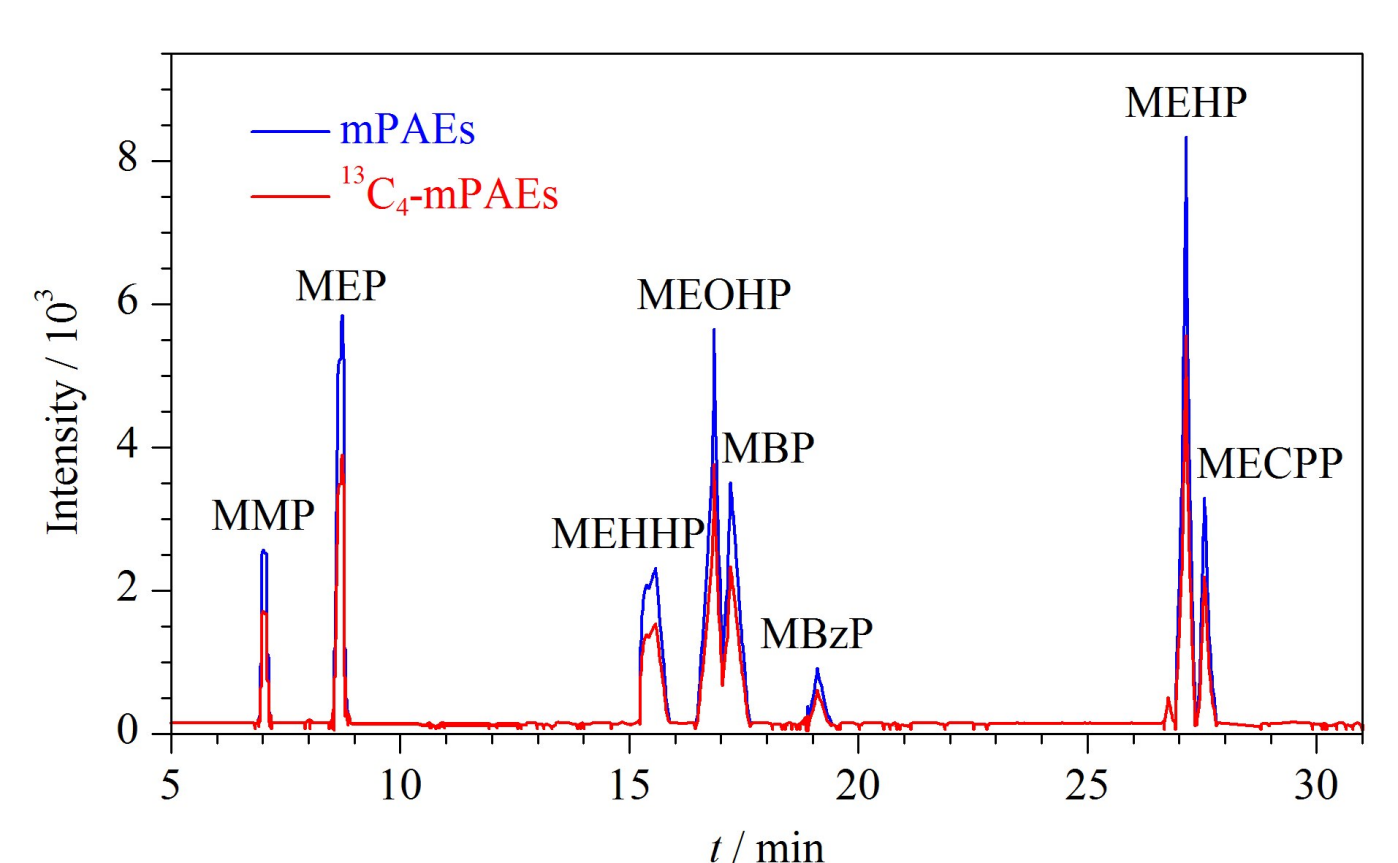
8种mPAEs和8种同位素内标的总离子流色谱图

### 2.2 方法学评价

#### 2.2.1 线性范围、相关系数、检出限和定量限

配制系列质量浓度(0.1、0.5、2、10、20、50、100、200 ng/mL)的8种mPAEs混合标准溶液并进样分析,以目标物与对应内标的定量离子峰面积之比为纵坐标(*y*)、目标物的质量浓度为横坐标(*x*, ng/mL),绘制标准曲线。结果如表3所示,8种mPAEs在0.1~200 ng/mL范围内线性关系良好,相关系数(*r*^2^)均≥0.993;以3倍信噪比计算检出限(LOD)、10倍信噪比计算定量限(LOQ), 8种mPAEs的LOD为0.015~0.048 ng/mL, LOQ为0.050~0.160 ng/mL。

**表3:** 8种mPAEs的线性范围、回归方程、相关系数、检出限和定量限

Compound	Linear range/(ng/mL)	Regression equation	*r*^2^	LOD/(ng/mL)	LOQ/(ng/mL)
MMP	0.1-200	*y*=5.19*x*-0.025	0.998	0.036	0.120
MEP	0.1-200	*y*=4.82*x*-0.190	0.997	0.048	0.160
MEHHP	0.1-200	*y*=13.15*x*+0.059	0.997	0.015	0.050
MEOHP	0.1-200	*y*=7.14*x*-0.055	0.997	0.016	0.053
MBP	0.1-200	*y*=4.64*x*+0.007	0.998	0.027	0.090
MBzP	0.1-200	*y*=13.29*x*+0.089	0.993	0.034	0.113
MEHP	0.1-200	*y*=4.22*x*+0.101	0.997	0.028	0.093
MECPP	0.1-200	*y*=1.14*x*+0.004	0.998	0.030	0.100

*y*: peak area ratio of mPAEs to internal standard; *x*: mass concentration, ng/mL.

#### 2.2.2 回收率与精密度

在空白尿液中分别添加低、中、高3个水平(1、10、50 ng/mL)的8种mPAEs混合标准溶液,进行加标回收试验,每个加标水平做6次平行实验,并计算加标回收率和相对标准偏差(RSD)。

结果如表4所示,8种mPAEs在3个加标水平下的加标回收率分别为82.8%~96.9%、80.2%~94.2%和84.6%~99.7%,对应的RSD分别为5.8%~16.9%、6.7%~15.9%和6.8%~18.0。实验结果说明,该方法准确、可靠,能够用于实际尿液样品的检测。

**表4:** 8种mPAEs在尿液中的加标回收率和相对标准偏差(*n*=6)

Compound	1 ng/mL			10 ng/mL			50 ng/mL	
	Recovery/%	RSD/%		Recovery/%	RSD/%		Recovery/%	RSD/%
MMP	94.0	9.6		81.3	8.2		94.7	7.9
MEP	90.8	5.8		94.2	11.0		98.9	6.8
MEHHP	96.9	12.8		80.3	15.9		92.8	18.0
MEOHP	86.8	16.9		82.5	13.2		97.7	12.5
MBP	92.3	12.8		89.1	11.4		92.9	6.8
MBzP	88.0	8.8		88.3	6.7		84.6	17.8
MEHP	82.8	11.8		81.6	10.6		89.6	12.1
MECPP	84.7	10.9		80.2	6.8		99.7	12.9

### 2.3 与其他方法的比较

胎儿的器官系统尚未发育完全,长期暴露于母体内的mPAEs环境中,会对其生长发育造成危害,因此对母体尿液中mPAEs的检测灵敏度与准确度要求较高。为了考察本方法的有效性,将本方法与其他文献方法进行比较。通过比较LOD和回收率,本方法具有更高的灵敏度和较好的回收率(表5),能够满足孕妇尿液中mPAEs的分析检测。

**表5:** 本方法与其他文献方法的比较

Compounds	Analytical method	Matrix	LODs/(ng/mL)	Recoveries/%	Ref.
MMP, MBP, MBzP, MEP, MECPP, MEHP, MEHHP, MEOHP	SPE-HPLC-MS/MS	urine	0.015-0.048	80.2-99.7	this work
MMP, MEP, MEHHP, MiBP, MnBP, MEOHP, MBzP, MCHP, MEHP	UPLC-MS/MS	urine	0.060-0.850	84.1-122.0	[12]
MMP, MEP, MiBP, MnBP, MEHP, MBzP, MCHP, MOP, DiNP, MiDP, MEHHP, MEOHP	UPLC-MS/MS	urine	0.200-0.900	86.4-106.0	[13]
DMP, DEP, BBzP, DBP, DiBP, DEHP, MMP, MEP, MBzP, MEHP, MBP, MiBP	SPE-GC-MS	urine	0.100-0.330	73.1-116.8	[14]
MiBP, MBzP, MEHP	ILs-DLLME-HPLC	urine	0.290-0.340	93.8-99.1	[14]

MiBP: monoisobutyl phthalate; MnBP: monoisobutyl phthalate; MCHP: monocyclohexyl phthalate; MOP: mono-*n*-octyl phthalate; DiNP: diisononyl phthalate; MiDP: monoisodecyl phthalate; DMP: dimethyl phthalate; DEP: diethyl phthalate; BBzP: butyl benzyl phthalate; DBP: dibutyl phthalate; DiBP: diisobutyl phthalate; DEHP: di(2-ethylhexyl) phthalate; ILs: ionic liquids; DLLME: dispersive liquid-liquid microextraction.

### 2.4 孕妇尿液中mPAEs的暴露水平研究

低相对分子质量的邻苯二甲酸酯(LMW)包括MMP、MBP、MBzP和MEP,用ΣLMW表示上述4种LMW的含量总和;邻苯二甲酸二(2-乙基己基)酯(DEHP)的4种代谢产物分别为MECPP、MEHHP、MEOHP和MEHP,用ΣDEHP表示上述4种代谢产物的含量总和;用ΣmPAEs表示8种mPAEs的含量总和。本研究共测定了497名孕妇尿液中的mPAEs水平,结果如表6所示。实验结果表明,8种mPAEs均有检出,中位数检出水平从大至小依次为MBP、MMP、MECPP、MEHHP、MEP、MEOHP、MEHP、MBzP; MBP的检出水平最高,其中位数水平为104.46 ng/mL,可能与当地居民广泛使用树脂和塑料产品有关;MBzP的检出水平最低,其中位数水平为0.22 ng/mL; ΣLMW的中位数水平为142.50 ng/mL, ΣDEHP的中位数水平为29.99 ng/mL, ΣmPAEs的中位数水平为181.90 ng/mL,这一结果与覃丹俞等^[15]^的研究结果相近。古小明等^[16]^也发现从事PAEs生产相关职业的暴露人群尿液中,以MEP和MBP的检出水平最高。MBP的高水平暴露可能与鄂州市近几年的聚氯乙烯化合物(PVC)工业生产有关,为了获得良好的性能,一些工厂会在PVC中添加增塑剂,其中MBP类增塑剂占产物总量的50%~60%^[17]^。

**表6:** 孕妇尿液中8种mPAEs的检出水平分布及检出率(*n*=497)

Compound	Detection rate/%	Percentile levels/(ng/mL)			Detection level range/ (ng/mL)
		25th	50th	75th	
MMP	100	4.50	10.17	22.88	0.18-408.38
MEP	100	3.33	8.04	18.84	0.18-908.23
MEHHP	100	4.11	8.55	15.73	0.36-202.88
MEOHP	100	3.70	7.66	14.36	0.28-180.62
MBP	100	33.88	104.46	254.69	0.17-1994.30
MBzP	90.1	0.15	0.22	0.37	<LOD-8.80
MEHP	100	0.61	1.93	4.92	0.18-206.75
MECPP	99.4	4.54	9.43	17.95	<LOD-364.11
ΣLMW	/	53.64	142.50	307.56	0.79-2071.63
ΣDEHP	/	15.80	29.99	55.22	1.06-619.96
ΣmPAEs	/	76.73	181.90	392.20	3.39-2158.56

LMW: low-molecular-weight phthalate; DEHP: di(2-ethylhexyl)phthalate; ΣLMW=MMP+MEP+MBP+MBzP; ΣDEHP=MEHHP+MEOHP+MEHP+MECPP; ΣmPAEs=MMP+MEP+MEHHP+MEOHP+MBP+MBzP+MEHP+MECPP; /: no value.

### 2.5 孕妇尿液中mPAEs水平与新生儿出生体重、出生身长和孕周之间的关系

由于尿液中mPAEs的水平呈偏态分布,本研究将mPAEs水平进行自然对数(ln)转换,使其呈正态分布,再采用线性回归分析方法来探究孕期mPAEs暴露与新生儿出生体重、身长和孕周之间的关系。在统计分析中,若代谢物水平低于LOD,则以LOD/ $\sqrt{2}$ 代替。

在对各个混杂因素进行调整后发现,孕妇尿液中的MBP每增加一个ln水平,新生儿的出生身长增加0.10 cm(95%置信区间(CI): 0.01~0.19),孕周增加0.13周(95%CI: 0.05~0.21);孕妇尿液中的MBzP每增加一个ln水平,新生儿的出生身长增加0.14 cm(95%CI: 0.01~0.28);孕妇尿液中的MEP每增加一个ln水平,孕周减少0.11周(95%CI: -0.18~-0.03);孕妇尿液中的MECPP每增加一个ln水平,新生儿的出生体重降低39.62 g(95%CI: -73.73~-5.52);孕妇尿液中的MEOHP每增加一个ln水平,新生儿的出生体重降低39.28 g(95%CI: -76.48~-2.09)。实验结果表明,MEHHP的暴露水平与新生儿的出生体重呈负相关趋势,MMP、MEP和MECPP与新生儿的出生身长呈负相关趋势,详细结果如图2所示。

**图2:**
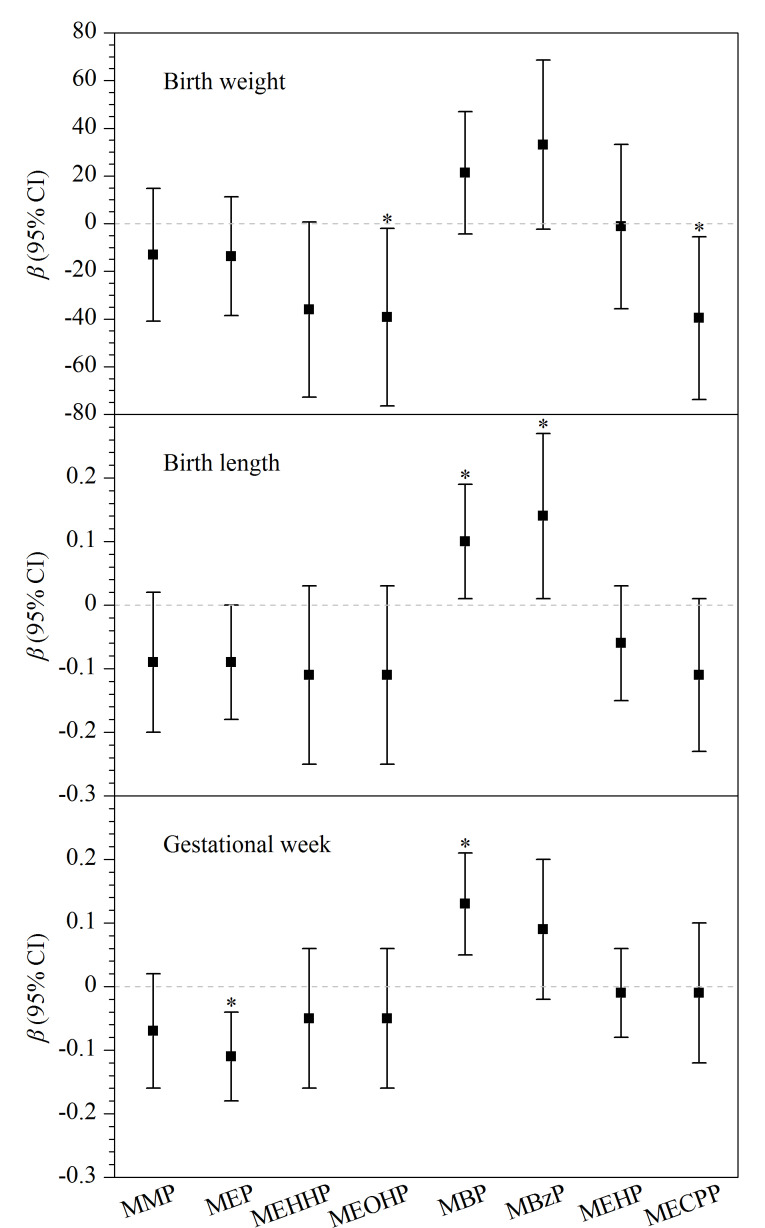
孕妇尿液中mPAEs水平对新生儿(a)出生体重、(b)身长和(c)孕周的影响(*n*=497)

高慧^[18]^发现孕妇尿液中MECPP的暴露水平与新生儿的出生体重呈负相关;孙小杰^[19]^研究发现,母亲静脉血与脐带血以及新生儿胎便中MBP与MEHP的暴露水平均与新生儿的低出生体重发生风险呈正相关。动物实验和体外实验证明,暴露于某些邻苯二甲酸单酯(如MBP和MBzP)可能会通过影响钠碘转运体和其他转运蛋白的功能,干扰细胞对甲状腺激素的摄取,进而影响甲状腺功能^[20,21]^,最终对胎儿的生长发育产生不利影响^[22⇓-24]^。本研究中发现MBP的暴露水平与新生儿身长和孕周呈正相关,这一结果与罗璨^[25]^和高慧^[18]^的研究结果一致,MBP对于新生儿身长和孕周的积极影响作用机制尚不明确,仍需用更多的动物实验和观察性实验来探究。除此之外,在本研究中MBzP的检出水平较低,而MBzP与新生儿身长呈正相关,可能是因为在较低水平时MBzP的剂量反应关系表现为积极作用。

## 3 结论

本研究建立了固相萃取-高效液相色谱-串联质谱同时检测孕妇尿液中8种mPAEs的分析方法,该方法检出限低,回收率及精密度良好,适用于环境污染物研究中大样本人群的生物监测。基于鄂州市妇幼保健院的497名孕妇尿液样本及新生儿出生结局,本研究发现孕妇主要暴露于MBP和MEP,且部分mPAEs会影响新生儿的出生体重、身长和孕周。这些结果表明,孕期mPAEs暴露会对新生儿的不良出生结局造成影响。后续需要更多大型队列研究来证实这些发现,同时利用动物模型或体外实验进行机理研究,以揭示mPAEs对不良出生结局影响的潜在生物学机制。
